# Spatial Epidemiology: Current Approaches and Future Challenges

**DOI:** 10.1289/ehp.6735

**Published:** 2004-04-15

**Authors:** Paul Elliott, Daniel Wartenberg

**Affiliations:** ^1^Small Area Health Statistics Unit, Department of Epidemiology and Public Health, Imperial College London, London, United Kingdom; ^2^Environmental and Occupational Health Sciences Institute and The Cancer Institute of New Jersey, University of Medicine and Dentistry of New Jersey, Robert Wood Johnson Medical School, Piscataway, New Jersey, USA

**Keywords:** disease clusters, disease mapping, environmental pollution, epidemiology, geographic studies, methods

## Abstract

Spatial epidemiology is the description and analysis of geographic variations in disease with respect to demographic, environmental, behavioral, socioeconomic, genetic, and infectious risk factors. We focus on small-area analyses, encompassing disease mapping, geographic correlation studies, disease clusters, and clustering. Advances in geographic information systems, statistical methodology, and availability of high-resolution, geographically referenced health and environmental quality data have created unprecedented new opportunities to investigate environmental and other factors in explaining local geographic variations in disease. They also present new challenges. Problems include the large random component that may predominate disease rates across small areas. Though this can be dealt with appropriately using Bayesian statistics to provide smooth estimates of disease risks, sensitivity to detect areas at high risk is limited when expected numbers of cases are small. Potential biases and confounding, particularly due to socioeconomic factors, and a detailed understanding of data quality are important. Data errors can result in large apparent disease excess in a locality. Disease cluster reports often arise nonsystematically because of media, physician, or public concern. One ready means of investigating such concerns is the replication of analyses in different areas based on routine data, as is done in the United Kingdom through the Small Area Health Statistics Unit (and increasingly in other European countries, e.g., through the European Health and Environment Information System collaboration). In the future, developments in exposure modeling and mapping, enhanced study designs, and new methods of surveillance of large health databases promise to improve our ability to understand the complex relationships of environment to health.

Spatial epidemiology is the description and analysis of geographically indexed health data with respect to demographic, environmental, behavioral, socioeconomic, genetic, and infectious risk factors. It is part of a long tradition of geographic analyses dating back to the 1800s when maps of disease rates in different countries began to emerge to characterize the spread and possible causes of outbreaks of infectious diseases such as yellow fever and cholera (Walter 2000). Over the ensuing decades, it grew in com.plexity, sophistication, and utility. Spatial epidemiology extends the rich tradition of ecologic studies that use explanations of the distribution of diseases in different places to better understand the etiology of disease (Doll 1980; Keys 1980). In this article we focus principally on small-area analyses of chronic, noninfectious diseases, where there is considerable current interest within the field of spatial epidemiology.

Recent advances in data availability and analytic methods have created new opportunities for investigators to improve on the traditional reporting of disease at national or regional scale by studying variations in disease occurrence rates at a local (small-area) scale (Walter 2000). Such investigations may include locally relevant health risk factor data such as exposures to local sources of environmental pollution and the distribution of locally varying socioeconomic and behavioral factors. They also present new challenges because as the scale of the investigation becomes narrowed to a particular small area or group of areas, the reduced size of the population at risk leads to small numbers of events and unstable risk estimates (Olsen et al. 1996). Furthermore, because of the small population, such studies are more susceptible to errors or local variations in the quality of both the health (numerator) and the population (denominator) data than studies conducted over larger areas. At the broader scale, purely local variations in data quality are likely to largely cancel out, whereas at the small-area scale, these variations could lead to serious biases if not detected. Finally, small-area studies (like other types of epidemiologic inquiry) are susceptible to confounding, which can result in spurious exposure–disease associations. In the small-area case, this is particularly so with respect to socioeconomic variables. People and communities tend to cluster in space in systematic ways that may be highly predictive of disease risk. For example, people of high socioeconomic status tend to live near others with high incomes and in areas with better housing and schooling than those in lower-income areas. Individuals with higher incomes tend to have more favorable risk factor profiles (e.g., they are more likely to be nonsmokers, take more leisure-time exercise, and eat more favorable diets) and as a consequence, have better health (Smith et al. 1996a, 1996b). Such spatially organized socioeconomic effects can have important influence on the rates of disease observed in small areas (Dolk et al. 1995). They may also be associated with the siting (or absence) of sources of environmental pollution, as “environmental (in)justice” dictates that poorer people in poorer areas are often more likely to be exposed to the effects of pollution (Corburn 2002).

We note that an in-depth and individual-based approach might investigate how individuals interact with their environment and how these interactions affect health. This could address, for example, why people with higher incomes take more leisure-time exercise. Is it because they have a local environment more enticing, have the financial resources to engage in specific activities, have jobs that afford them more leisure time, or pursue more leisure-time activities for other reasons? Such questions may have an important spatial component. However, we see these as second-order issues beyond the scope of this article.

We now briefly consider the analytic framework for carrying out spatial analyses and the types of studies commonly undertaken. We then focus on a number of challenges that face the practitioner of spatial epidemiology, including issues of data availability and quality, confidentiality, exposure assessment, exposure mapping, and study design.

## Analytic Framework

In considering an analytic framework for spatial epidemiologic analyses (Elliott et al. 2000b), we first distinguish between point and area data. Each of the population, environmental exposure, and health data may be associated with a point, or exact spatial location such as a street address (occurrence data), or an area, a defined spatial region such as a community, of which it is representative (aggregate summaries, e.g., count data). Data from a variety of points (e.g., residence, workplace, hobby locations) may give the closest link to an assumed biologic model in which the average disease risk of an individual will reflect individual characteristics such as age, sex, and genetic factors (e.g., predisposition, susceptibility, immune or toxicologic response capability); lifestyle variables, such as smoking and diet; and exposure to environmental pollutants. The lifestyle and exposure factors may depend on the ways that the individual interacts with the environment as she/he moves through both time and space, which itself depends on the range of daily activities, type and location of residence, workplace, travel and migration patterns, habits, behaviors, and so on. Together with individual susceptibility factors, these may determine biological dose. For many environmental exposures, the parameter of interest may be cumulative lifetime dose, the maximum short-term dose, or even the cumulative dose above some threshold. For example, in carcinogenesis, the parameter may be the dose at some critical point in the multistage pathway underlying cancer formation (Moolgavkar 1999). For other outcomes, exposure to a single, high (toxic) dose may trigger an adverse response, as with chloracne after exposure to 2,3,7,8-tetrachlorodibenzo-*p*-dioxin (TCDD) from the Seveso accident in northern Italy (Caramaschi et al. 1981). The effects from ionizing radiation, on the other hand, are thought to reflect cumulative lifetime exposure, a more problematic metric for spatial epidemiology, although recent research suggests that the maximum rate of exposure mediates the effects (Cardis et al. 2001).

Case–control and cohort studies can give a relatively close approximation to the biologic model in investigating environmental health issues because both individual person characteristics and exposures are studied in the individual environment. Case–control studies provide point data for cases and a set of controls. They are prone to selection and other biases, are moderately expensive and time-consuming to carry out, and are not feasible in all situations. Cohort studies, although not subject to selection bias, are prone to other biases, including losses to follow-up, and are generally more expensive and time-consuming to carry out than case–control studies. Exploratory studies using aggregate data, such as geographic correlation studies, offer an alternative approach for generating, prioritizing, and analyzing data to address specific hypotheses of disease etiology and causation. Although they too are prone to biases and misclassification (Elliott and Wakefield 2000), they are generally easier, quicker, and less expensive to conduct than case–control or cohort studies. One example of this approach is with use of a dedicated system such as that developed by the Small Area Health Statistics Unit (SAHSU) in the United Kingdom (Elliott et al. 1992b); this has recently been adopted in other European countries as part of the European Health and Environment Information System (EUROHEIS) collaboration (EUROHEIS 2003). If these exploratory and other studies generate sufficient evidence in support of specific hypotheses, case–control and/or cohort studies can then be used to test these hypotheses with use of purpose-collected individual-level data.

## Types of Spatial Epidemiologic Inquiry

Spatial epidemiology at small-area scale can be divided into three main areas:

- disease mapping
- geographic correlation studies
- clustering, disease clusters, and surveillance.

We note that the above grouping is artificial. For example, depending on scale, disease mapping may provide information on individual disease clusters and more generally on disease clustering. A point source of exposure may give rise to a localized excess of cases that might be detected on a disease map, whereas geographic correlation studies share much in common with disease-mapping studies (with addition of one or more potential explanatory variables), and the statistical models used are often similar. Each of the above main types of inquiry is now considered in turn.

### Disease Mapping

As noted earlier, disease maps have a long history. A survey in 1991 identified 49 international, national, and regional disease atlases (Walter and Birnie 1991). An early example was the work of Stocks, who described variations in cancer mortality across counties of England and Wales (Stocks 1936, 1937, 1939). More recent examples include an atlas of cancer incidence in England and Wales (Swerdlow and dos Santos Silva 1993) and an all-causes mortality atlas (Pickle et al. 1996) and separate cancer mortality atlas (Devesa et al. 1999) for the United States. Disease maps provide a rapid visual summary of complex geographic information and may identify subtle patterns in the data that are missed in tabular presentations. They are used variously for descriptive purposes, to generate hypotheses as to etiology, for surveillance to highlight areas at apparently high risk, and to aid policy formation and resource allocation. They are also useful to help place specific disease clusters and results of point-source studies in proper context (Wilkinson et al. 1997).

Disease maps typically show standardized mortality or morbidity (e.g., incidence) ratios (SMRs) for geographic areas such as countries, counties, or districts. The rate in area *i* is estimated by the standardized mortality (or morbidity) ratio (SMR*_i_*), calculated as *O_i_* /*E_i_*, where *O_i_* is the observed number of deaths or incident cases of disease in the area (assumed to follow an independent Poisson distribution). *E_i_* is the expected number of cases (calculated by applying age- and sex-specific death or disease rates to population counts for the area). The SMR thus defined is based on indirect standardization. Some authors advocate direct standardization, as it involves adjustment to a common standard (Julious et al. 2001). In our own experience, the two methods nearly always give near-identical results.

**Figure 1:**
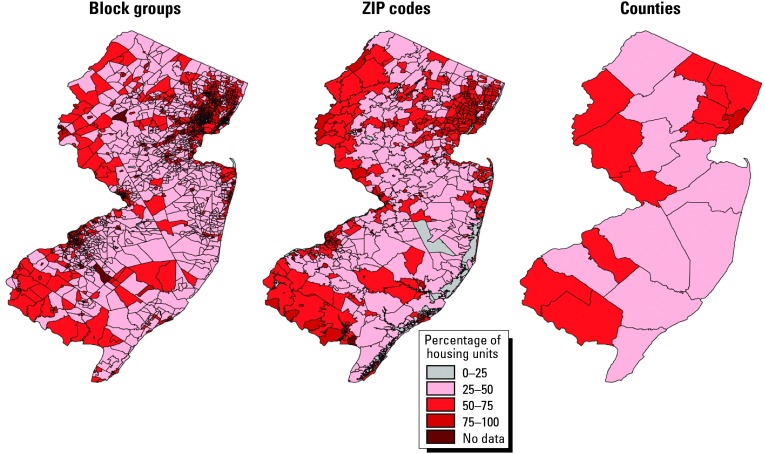
Percentage of homes built before 1950 in New Jersey based on U.S. census data reported at the block group level of resolution. The three maps depict the same data at three different scales: U.S. census block group, ZIP codes, and counties.

Although disease maps have both visual and intuitive appeal, caution is required in interpretation, as apparent patterns can be created or lost artifactually depending on how the mapped variable is depicted (e.g., the number and boundaries of the categories) and the geographic scale or resolution. The choice of colors for displaying data can also affect interpretation (Brewer and Pickle 2002; Smans and Esteve 1992). Maps of the same data drawn at different scales of resolution can result in very different visual patterns (Monmonier 1997). Figure 1, for example, from a study of childhood lead poisoning, shows maps at three different scales (U.S. census block group, ZIP codes, and counties) of the percentage of homes built before 1950 (a major risk factor for childhood lead overexposure) in New Jersey based on U.S. census data reported at the block group level of resolution. When aggregated by geopolitical boundaries, regional values are overweighted (geographically) by more compact, more urban ones that typically have more older housing, often obscuring important information in less-populated rural regions.

When constructing maps, users must select both the size of units and the method to aggregate units to highlight the features of interest. Homogeneity within aggregate groups is important for meaningful interpretation. Different scales and different aggregation strategies can lead to different but equally valid maps that emphasize different features of the data. In the geography literature, this is called the modifiable area unit problem (Openshaw 1984). Although generally the aim is to choose geographic units that are as small as possible, the choice is often dictated by the availability of data, and because of sparse data, there will often be a tradeoff between homogeneity within small geographic units and precision of risk estimates.

Variation in rates across the map may reflect differences in the quality of data, for example, in the diagnosis, classification, or reporting of disease (Best and Wakefield 1999), rather than true differences in disease rates. Furthermore, the digital boundaries identifying the geographic units, and the geographic linkages between the various data within a geographic information system (GIS) may contain errors, including errors in the assignment of geocodes (postcodes) (Briggs and Elliott 1995). Clearly these may lead to errors in the resultant maps. Data quality for denominator (population at risk) data, although often overlooked, can also be a problem. Inaccurate estimates can change the appearance of mapped patterns and complicate map comparisons, especially for areas with small populations. When calculating SMRs for intercensual years, investigators use different interpolation algorithms, which can lead to differences in denominators and rates. For example, in a study of cancer incidence in Dalgety Bay, Scotland, risks based on census data were overestimated because there had been rapid population growth in the area since the previous census (Black et al. 1994).

Recent focus on small-area mapping studies, where typically the unit of analysis has a population of 5,000 or less (such as census tracts in the United States or electoral wards in the United Kingdom), introduces an extra source of variability into the map because of random variation. Typically, sparsely populated areas with few (or zero) cases can generate extreme values of the SMR, as the variance of the SMR is inversely related to *E_i_* and small populations will have large variability in the estimated rates. As these sparsely populated areas are often bigger than densely populated areas (because the administrative geography depends on population size), they tend to dominate the map visually even though they produce the least-precise risk estimates (Elliott et al. 1995). Methods based on Bayesian statistics (Clayton and Kaldor 1987) have been used to remove part of the random component from the map to give smoothed estimates of relative risk in each area. Such estimates are a compromise between the local value of the SMR and either the mean value for the map as a whole, or some local mean. Smoothing is greatest for the least-stable estimates (i.e., where *E_i_* is small).

**Figure 2:**
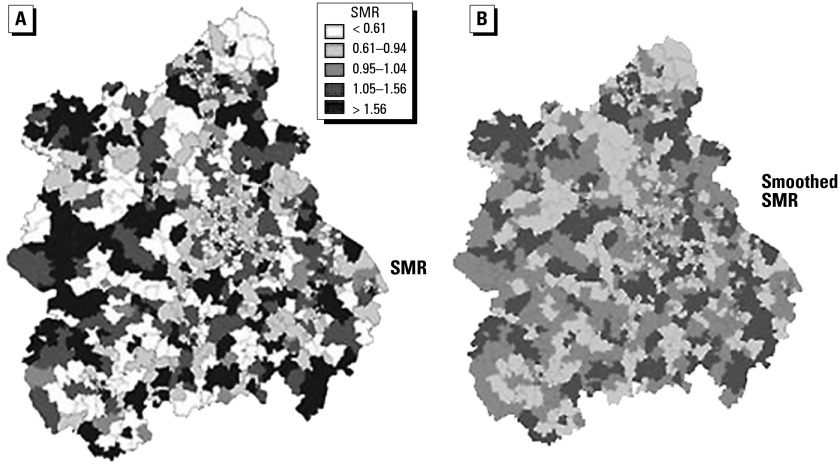
Adult leukemia by electoral ward in West Midlands Region, England, 1974–1986. (*A*). SMR; West Midlands = 1.0. (*B*) SMR after smoothing using empirical Bayes methods. Figure reproduced from Olsen et al. (1996), with permission of the BMJ Publishing Group.

Figure 2 is an example of a small-area mapping study of adult leukemia incidence in the West Midlands region of England, 1974–1986 (Olsen et al. 1996). Each small area on the map is an electoral ward, which as noted above has a population of approximately 5,000 on average. The smallest wards, with the largest populations and hence the most stable risk estimates, are located toward the center of the map in and around the Birmingham conurbation. Figure 2A shows the age- and sex-adjusted SMRs based on the observed and expected values in each area, whereas Figure 2B shows the smoothed SMR, with smoothing to the overall mean using empirical Bayes methods. The unsmoothed map has considerable apparent variability, with more than 3-fold variation across the map. Many of the extreme values (both low and high) are found in the periphery of the map, that is, in the rural areas distant from the Birmingham conurbation. After smoothing, the map appears much flatter, and all the extremes are removed.

Although map smoothing on average produce a more stable and realistic map, an important issue is the extent to which disease excesses in any truly high-risk areas (especially those more sparsely populated) might be smoothed away. The degree of smoothing will determine the tradeoff between high sensitivity (truly high-risk areas correctly identified) and high specificity (areas without excess risk correctly identified). This tradeoff is important, as a sensitive but nonspecific measure will generate many false positive findings, whereas a specific but nonsensitive measure will miss areas with high risk. Richardson et al. (2004) have investigated the properties of commonly used map-smoothing techniques using a series of realistic scenarios to simulate possible patterns in the disease map. They conclude that unless the relative risk is of the order of 2 to 3 and expected numbers in the geographic unit are at least 5 (or for relative risks of order 2, expected numbers are at least 20), then the map-smoothing methods are likely to perform poorly in terms of their abilities to detect areas with true excess. This is important in designing appropriately powered investigations and in managing expectations as to what can be achieved with sparse data.

### Geographic Correlation Studies

In geographic correlation studies, the aim is to examine geographic variations across population groups in exposure to environmental variables (which may be measured in air, water, or soil), socioeconomic and demographic measures (such as race and income), or lifestyle factors (such as smoking and diet) in relation to health outcomes measured on a geographic (ecologic) scale. This approach often takes advantage of data that are routinely available and can be used to investigate natural experiments where the exposure has a physical basis (e.g, soil, water) (Richardson and Monfort 2000). In addition, the effect of exposure measurement error is reduced by averaging across groups. However, geographic correlation is affected by the problems of disease-mapping studies noted above, together with the added complication of correlation with one or more explanatory variables. Such studies are often thought of as hypothesis-generating, as the unit of observation is the geographic group rather than the individual and associations observed at the group level do not necessarily hold at the individual level—the so-called ecologic fallacy (Piantadosi et al. 1988). For this reason, observations at the ecologic scale will usually need validation and replication at the individual level, for example, through cohort, case–control studies or possibly randomized, controlled prevention or intervention trials (such as lead chelation studies). Nonetheless, ecologic studies of this kind have been pivotal in developing and exploring major hypotheses of public health importance, for example, the linking of malignant hepatoma (which has very high incidence in Asian populations) with hepatitis B infection (Beasley 1988) and the seminal work of Keys and colleagues in elucidating the role of saturated fat in the etiology of coronary heart disease (Keys 1980).

The development of the first cancer mortality atlases in the United States in the mid-1970s (Mason et al. 1975, 1976) showed distinctive patterns of variation of different cancers and led to a series of informal correlational studies. Based on the patterns of high risk that appeared to correspond to specific activities, behaviors, or environmental exposures, investigators postulated specific hypotheses (Blot and Fraumeni 1982; Fraumeni 1988; Hoover et al. 1975; Mason 1976) that were later investigated through case–control studies. Although not all of these studies confirmed the geographically generated hypotheses, investigation of a regional excess of oral cavity and pharynx cancer among women revealed the previously unknown risk of smokeless tobacco use (Blot and Fraumeni 1977; Winn et al. 1981). Investigation of a regional excess of sinonasal cancer was consistent with studies in other countries showing risks associated with working in the furniture industry (Blot and Fraumeni 1977; Brinton et al. 1976, 1977, 1984, 1985), and study of local lung cancer excess was associated with residence near or employment in the arsenic industry (Blot and Fraumeni 1975, 1994).

Geographic correlation studies are also carried out at a more local or small-area scale, where the problem of ecologic bias may be lessened as the analysis is closer to the level of the individual. For example, Staessen et al. (1999) examined the relationship between environmental exposure to cadmium and bone density in 10 districts in Belgium (including 6 that bordered on three zinc smelters). Shaper et al. (1980) investigated the relationship between water hardness and cardiovascular disease in towns in Great Britain, while Maheswaran et al. (1999) assessed in particular the role of magnesium in the water supply in relation to mortality from acute myocardial infarction. The last of these studies used water zones in northwest England (each water zone serves up to 50,000 people) as the unit of analysis. For some environmental exposures, such as non-ionizing radiation from overhead power lines, the potential harmful effects may operate over a very small distance (up to 50–100 m from the power line), so only a highly localized or individual-based study can investigate the issue (Feychting and Ahlbom 1993; Olsen et al. 1993; Verkasalo et al. 1993).

One important issue merits brief mention here. Informal geographic correlation studies (or evaluations) are often conducted by non-scientists in their own communities or neighborhoods out of personal concern. When one suspects a local disease excess, or when oneself, a family member or friend is stricken with cancer, one often asks “Why? What did I or they do wrong? What is it about where I live or where I work that caused this tragedy?” This concern may cause one to seek an explanation or to consider local industries or sources of environmental pollution as the putative cause. In this process, an informal geographic correlation is being undertaken, insofar as the health event and putative environmental exposure have been juxtaposed. Most such evaluations do not provide useful etiologic clues, as neither the underlying variability in disease rates nor the post hoc nature of the association with sources of environmental pollution are properly accounted for.

### Disease Clusters, Clustering, and Surveillance

Investigation of disease clusters and disease incidence near a point source usually assumes that the background risk surface is flat, against which a peak at the pollution source is being tested. If this is not the case and the background surface is bumpy, that is, there are peaks and troughs in the risk surface, this may indicate generalized or broad-scale clustering of the disease. (Clearly in this situation, the observation of a disease excess at a particular point may not be unusual.) This tendency for disease cases to occur in a nonrandom spatial pattern relative to the pattern of the noncases has a more robust statistical formulation than the investigation of disease clusters per se and may give clues as to etiology (Wakefield et al. 2000). For example, there is evidence of spatial clustering of Hodgkin disease (Alexander et al. 1989) that, along with other epidemiologic and laboratory evidence, has suggested a possible infectious etiology. The study of generalized clustering has much in common with disease mapping, and the same cautionary considerations apply, particularly concerning the quality of the underlying data.

Putative disease clusters may come to light because of media reports or be brought to the attention of the authorities by concerned individuals; as noted, often the apparent cluster will become linked with a local source of environmental pollution (Greenberg and Wartenberg 1991; Trumbo 2000). In general, this might be a point, line, or area source. Point sources include a chimney stack from an industrial site, a radio transmitter, mobile phone tower, and so forth. A line source refers to an extended linear source such as a road, river, or power line, and an area source may include industrial complexes, landfill sites, and other geographically defined areas such as water-supply zones (or watersheds). In practice, in the absence of detailed information concerning the extent of an industrial site or the locations within the site where emissions occur, area sources are often modeled as point sources. A recent study of landfill sites in the United Kingdom would be one example (Elliott et al. 2001). Although U.S. case–control studies have used similar exposure metrics, no extant systems allow similar, broad-based data assessments.

The term disease cluster is poorly defined but implies an excess of cases above some background rate bounded in time and space. These boundaries may be ill-defined, and so-called boundary shrinkage may occur, accentuating the apparent risk by focusing the investigation tightly on the cases making up the cluster.

The more narrowly the underlying population is defined, the less will be the number of expected cases, the greater will be the estimate of the excess rate, and often the more profound will be the statistical significance. (Olsen et al. 1996)

Despite the inherent problems, the local public health department may find itself compelled to respond, if only to allay public anxiety (Greenberg and Wartenberg 1991). Usually the initial assessment of the data will involve the following:

- Detailed checking of the cases. This is an essential step, as the putative cluster may involve a disparate group of diagnoses, some double-counting (duplicate records) may occur, and some cases may be erroneously reported. One also must verify the location (or geocode) of each case, which can be difficult in some locales.
- Definition of the boundaries in time and space so that a population denominator, by age and sex, can be constructed (usually from census records). Although accuracy is important, it is hard to validate the population data outside the census years, particularly as the areas get smaller.
- Estimation of the expected numbers of cases based on age- and sex-specific background rates (e.g., obtained from published regional or national data).
- Calculation of the SMR for the area.
- Assessment of statistical significance (usually reported at *p* < 0.05) assuming a Poisson distribution for the occurrence of cases.
- Communication of results to the public, providing context, plausibility, and plans for follow-up, if appropriate.

The process of obtaining the initial data outlined above can be extremely costly in both time and resources for local health department personnel, as data from several disparate sources must be assembled and brought together. In addition, for local health departments not familiar with the detailed methods and requirements of a major cluster investigation, inevitably there can be a steep learning curve. This might include familiarizing oneself with the specialist statistical methodologies of cluster investigation (beyond calculation of the SMR), as such methods are not part of the routine armory of the public health specialist (Elliott et al. 1995; Morris and Wakefield 2000; Waller and Lawson 1995). In the United Kingdom, a rapid inquiry facility (RIF) has been established within SAHSU to provide such analyses within a few working days for a particular area. This greatly facilitates the ability of a local public health department to respond quickly to reports of a putative disease excess in their area based on the available routine data. Areas can be defined by administrative geography such as electoral enumeration district (~ 400 individuals) or ward, by post-code (~ 13 households), or by map reference. The RIF includes routine national health and population data held in an Oracle database on its own dedicated computer system, with geographic linkages provided by a proprietary GIS (Aylin et al. 1999). The health records, including mortality, cancer incidence, hospital discharges, and congenital anomalies, all include the postcode, with geographic resolution of approximately 10–100 m. The RIF assembles the data and provides an SMR (with and without adjustment for socioeconomic variables) for the area of interest compared with regional or national rates. An unsmoothed and smoothed map (using empirical Bayes methods) are also produced, together with contextual maps of local landmarks, socioeconomic data, pollution sources, and so on. A version of the RIF has been made available to other European countries as part of the EUROHEIS consortium (EUROHEIS 2003). Although many state health departments in the United States routinely evaluate data in response to cluster inquiries, none currently has a comparable system dedicated to such activities.

Once a link between a putative disease cluster and a local source of environmental pollution has been put forward, it is extremely difficult to confirm or refute it without recourse to external data (e.g., from another area or time period). Because an informal process of data comparison (akin to multiple testing) has taken place (by the media, concerned individuals, etc.) in similar-sized localities elsewhere across the country, statistical testing in a formal sense is rendered invalid (Elliott and Wakefield 2000). Only disease occurrences at the high end of the distribution are highlighted. Diseases or areas with apparent low risk never come to the attention of the authorities. This informal process of multiple testing means that it is impossible to gauge the true significance (in a statistical sense) of an apparent disease excess in a particular locality. Many clusters, even where nominally statistically significant, will appear purely as a chance finding, particularly for rare events (such as most cancers). Conversely, some true disease excesses may be overlooked because of lack of systematic evaluation of the small-scale geographic pattern of disease incidence (Wartenberg 1995).

Local concerns about a disease cluster in a particular area must be sympathetically and sensitively handled but will not usually lead to formal study or any new etiologic insight (Drijver and Woudenberg 1999; Trumbo 2000). Indeed, against this background, it has been argued that individual cluster reports should not be investigated (Rothman 1990) unless there are sufficient numbers of cases (five or more) and risks in a particular area are very high (relative risk ≥ 20) (Neutra 1990).

Occasionally it will be necessary to carry out more detailed inquiry. Investigations have adopted either the case–control (e.g., Aschengrau et al. 1998; Infante-Rivard and Amre 2001; Morris and Knorr 1996; Mulder et al. 1994; Wrensch et al. 1999) or small-area (ecologic) approach (e.g., Berry and Bove 1997; Goldberg et al. 1995; Kokki et al. 2001; Lopez-Abente et al. 2001; Wilkinson et al. 1997). Where the routine health statistics appear to confirm suspicions of disease excess (notwithstanding the problems of multiple testing referred to above), then as indicated, examination of data for a different time period or area will be required. This allows the data to be tested within the usual statistical paradigm, as the initial observation generates a hypothesis that can then be tested on independent data. With a dedicated national system such as SAHSU in the United Kingdom, this can be done readily using the national database. Examples include national studies of cancer incidence near incinerators of waste solvents and oils after observations of excess incidence of cancer of the larynx near one such incinerator (Elliott et al. 1992a), and risk of leukemia and incidence of other cancers near TV and radio transmitters, after reports of a leukemia cluster near the Sutton Coldfield transmitter in the West Midlands, England (Dolk et al. 1997a, 1997b).

When the study is done because of *a priori* concerns about a source of environmental pollution rather than in response to a claim of disease excess in a particular area, the statistical framework is again more robust, as a hypothesis can be set up and tested in the usual way. Investigation may involve a number of or all such sources in the region or country. This increases statistical power and overcomes the problem of selection where one site, or a few sites, are chosen for study, perhaps because of suspicion of disease excess in the vicinity. However, it makes the possibly unrealistic assumption that the sources are similar with respect to their potential to cause environmental health problems, and high risk around one or two sources (which may have high levels of toxic releases into the environment) may be masked. In the United Kingdom, national studies undertaken *a priori* include cancer incidence near municipal incinerators (Elliott et al. 1996a), risk of hemopoietic cancers near oil refineries (Wilkinson et al. 1999), angiosarcoma of the liver near vinyl chloride plants (Elliott and Kleinschmidt 1997), and risk of congenital anomalies and various cancers near landfill sites (Elliott et al. 2001; Järup et al. 2002). In the Scandinavian countries, national studies of leukemia risk near power lines have been done that take advantage of the high-quality health and population registers available in those countries (Feychting and Ahlbom 1993; Olsen et al. 1993; Verkasalo et al. 1993).

Although national-scale small-area studies are unlikely on their own to establish causal links with the pollution source (unless the risk is very high), they do give a valuable answer to the public health question “If I live near polluting source X, am I (on average) at increased risk of disease?” and may indicate avenues for further inquiry such as studies of pathways of environmental exposure, biomarker studies, or case–control studies.

#### Cluster detection and surveillance.

Surveillance, or the systematic routine collection and analysis of health outcome data for disease prevention and control purposes (Thacker and Berkelman 1992), can be applied to the problem of disease clusters through the use of space, time, and space-time pattern detection methods (Kulldorff et al. 1997; Kulldorff 1997, 2001; Rogerson 1997, 2001; Rushton et al. 1996). This has been proposed as a more effective approach than ad hoc cluster studies for identifying local disease excesses and prioritizing them for follow-up investigations (Hardy et al. 1990; Wartenberg 1995). In contrast to the passive or reactive analysis of reported local disease excesses using systems like the RIF, surveillance offers the opportunity to provide proactive, early detection of raised incidence of disease even when there is no specific etiologic hypothesis. In addition to increasing the likelihood of identifying etiologic clusters, which may implicate behavioral, environmental contamination or other preventable risk factors, this approach could enable public health officials to identify potential problems earlier and conduct preliminary evaluations of nonetiologic situations that may be of concern to the public. In so doing, the officials would be able to respond to inquiries in a more thorough, consistent, scientific, and timely manner. This is in contrast to the current situation with disease clusters, already noted, where most potentially hazardous problems are investigated only after local residents, physicians, or others have brought them to the attention of health officials, often through political pressure or media publicity. A proactive identification system could also enable more timely interventions where warranted, ranging from education to increased screening to environmental cleanup, and more rapid assessment and possible resolution of community concerns when there is a valid, alternative explanation to the perception of a disease excess.

Proactive surveillance systems have been effective for disease prevention and control when applied to infectious disease outbreaks, occupational exposures and disease (Dubrow and Wegman 1983; Whorton et al. 1983; Williams et al. 1977), and adverse reactions to pharmaceuticals (Strom 2000) (often termed postmarket drug surveillance). Similar systems for the assessment of acute outbreaks have been developed and implemented in response to concerns about outbreaks from biological, chemical, or radiologic terrorism in which rapid, scientific assessment is essential for protecting the public health (Das et al. 2003; Gesteland et al. 2003; Platt et al. 2003).

Data quality issues are again important, as detecting apparent local clusters of disease may merely indicate areas with higher-quality data registration or perhaps areas of poor data quality where there are many duplicate registrations. Specificity is also a major issue, as, given the size of the database, the range of diseases, different age and sex strata, myriad definitions of areas of various sizes and configuration, and so forth, many false-positive clusters are bound to occur. For a surveillance program to be efficient and effective, researchers must provide methods for discrimination of true alarms, false alarms (false positives), and those situations that are less clear or equivocal, so that health department officials would not be obliged to follow up all apparent aberrations. One possible approach is to survey potential local sources of risk for the specific disease in question as is done currently for many cluster reports and respond only if there is an independent source of confirmatory or consistent environmental evidence. For those disease excesses for which there is a plausible, nonenvironmental explanation, clear and thoughtful communication to concerned communities based on solid scientific evidence could help dispel their urgent concerns.

For these reasons, in common with most public health departments, we do not currently advocate carrying out surveillance for chronic disease excesses as a matter of public health practice. We believe that this type of surveillance should not be put into practice until such time as the underlying data and methodologies provide a robust framework to support this activity, as would be the requirement for screening for other public health concerns. Nonetheless, we believe that development and evaluation of surveillance approaches is an important and priority area for future research on disease clusters.

## Challenges

### Data Availability and Quality

To carry out small-area studies using routine data sources, the basic data need to be made available, with high quality, and the inclusion of a geographically referenced code, such as the postcode in the United Kingdom or the census block or block group in the United States. Data should include (at the least) cancer registration as well as mortality, natality, and population data. Although natality and mortality data are a statutory requirement in developed countries, not all countries (including the United States) have a national cancer registry, reducing the ability to carry out studies of environmental health problems. In the United States, the Centers for Disease Control and Prevention (CDC) has established a program in environmental public health tracking, one component of which funds states to develop additional registries of health outcomes, such as asthma, for assessment of possible environmental etiologies (http://www.cdc.gov/nceh/tracking).

In purpose-designed case–control studies, detailed evaluation of the health data and assessment of the quality of the diagnostic information (for example, case note and histology review) are likely. In contrast, for spatial epidemiologic studies that rely on routine data sources, it is usually not possible to carry out detailed validation studies of the database. However, some assessment of the basic quality of the routine data is essential to inform their use in spatial analyses, and some limited validation of the cases might be undertaken (Elliott et al. 2000a). As already noted, the denominator data may contain substantial errors, particularly in the inter-censual years at small-area scale, and for the health event data there is always the potential for diagnostic error or misclassification, especially at older ages where diagnostic tests and postmortem examinations are carried out less frequently than at younger ages. Some events may be captured poorly, if at all, in routine registers (e.g., early abortions). For others, such as cancers, case registers may be subject to double counting and underregistration as well as diagnostic inaccuracies (Best and Wakefield 1999).

One type of relevant data not readily available in the United States or the United Kingdom is the history of residential locations. For longer-latency health outcomes, such as cancer incidence and many types of mortality, knowing the residential history of an individual would be far more useful for reconstructing exposure histories than his/her location/residence at time of diagnosis or death. Even for natality data, it has been shown in small studies in both the United States and the United Kingdom that between 20 and 25% of women change residences between date of conception and delivery (Khoury et al. 1988; Nelson 2003; Shaw and Malcoe 1991). However, as many move to nearby addresses (Nelson 2003), residential exposures may not change too much.

In contrast, the Scandinavian countries maintain historical registries of residences, and these have proved invaluable, as in the example already noted of constructing exposure histories to low-frequency electromagnetic fields from overhead power lines (Feychting and Ahlbom 1993; Olsen et al. 1993; Verkasalo et al. 1993). In the future, these types of data might become available in the United Kingdom through linkage to the National Health Service (NHS) number, although there are confidentiality issues concerning use of these data. In the United States, census data provide limited migration data to and from areal units, but typically data are not available for individuals. Although knowing when and where disease occurred is useful, knowing when and where prior exposures occurred is crucial for investigating etiology.

In the future, the increasing use and availability of computerization in medical care means that large new databases of morbidity, linked to individuals, may become available. Examples include general practitioner consultations in the United Kingdom, whereas in the United States there is particular interest in syndromic surveillance (e.g., Hartman et al. 2004). The quality of such data will need careful evaluation and no doubt will vary across specialties and medical practice and over time and space. Nonetheless, they promise exciting new opportunities for carrying out spatial epidemiologic inquiries using softer end points than those currently available, and hence potentially increasing the sensitivity of the methods to detect environmental health problems.

### Data Protection and Confidentiality

The current climate of legislation in the United States and the European Union is providing greater recognition of the rights of individuals to confidentiality of personal data, including health data, and the need for consent for medical investigations. In 2003, the United States brought into force the Privacy Rule (Department of Health and Human Services 2002) arising from the Health Insurance Portability and Accountability Act of 1996 (1996) that further complicates this issue. This potentially impinges on the secondary use of routine data for epidemiology (including spatial epidemiologic studies) where the data were originally collected for other purposes (e.g., health care management or delivery), but consent for their use for medical research is impracticable. In the United Kingdom, recent legislation has made it possible to use such routinely collected data without consent if certain conditions and safeguards are met. It is imperative for the future of epidemiologic research that such uses of the data are allowed to continue, provided that appropriate safeguards are in place.

In addition, with the recent increase in availability of fine-scaled, geocoded data, there is a new concern about the confidentiality of blocks, neighborhoods, and communities. The ability to acquire data and map high rates of adverse outcomes, clusters, or areas with high levels of pollutants can cause concern and outrage and possibly influence property values. Yet, rules and principles of good practices for analysts and others are still in the formative stages. Providing researchers access to these data is necessary for this field of research to progress, but implementing appropriate controls for confidentiality and protection of data is essential to maintain the trust and support of the public.

### Exposure Assessment, Exposure Mapping, and Study Design Issues

The quality of the exposure data has been regarded as the Achilles heel of environmental epidemiology. This holds true for spatial epidemiology, where distance is often used as a proxy for exposure to environmental pollutants, or some other geographic measure is used, for example, plume modeling (Nyberg et al. 2000). Although the availability of GIS has greatly enhanced the capability for spatial interpolation of exposure data (Briggs and Elliott 1995), the quality of the mapping depends on the accuracy and representativeness of the available input data, as well as the inherent validity of the interpolation method used.

Such approaches may provide valid first-order approximations to group or population exposure but may not capture individual exposure well nor allow for individual variations in absorption and susceptibility. Poorly measured exposure data can produce differential errors leading to systematic bias or result in random errors or imprecision, which (unless corrected) typically lead to bias toward no effect (Bernardinelli et al. 1997). More generally, such geographic methods of exposure assessment make a number of key assumptions that may limit their applicability in given situations (Elliott and Wakefield 1999). These include the following:

- equating environmental exposure (i.e., external to the individual) with biologic (internal) dose
- equating current exposure with past exposure
- equating modeled estimates of exposure (including distance-based measures) with true exposure
- equating exposure at a point (e.g., place of residence) with total personal exposure, that is, exposure integrated over the course of daily activities as the individual moves through the exposure field
- equating group exposure and group exposure–disease relationships with individual exposure and relationships at the individual level, that is, ecologic fallacy (Piantadosi et al. 1988).

An important issue in geographic analyses is the extent that the population of the areal unit is homogenous, both with respect to the environmental exposure under investigation and potential confounders. Within-unit variability in these factors could lead to bias in risk estimates (Elliott and Wakefield 2000). Recently, interest has focused on semiecologic designs that combine data on the general population with individual-level survey data (Plummer and Clayton 1996). For example, the INTERSALT study, a cross-sectional study of over 10,000 people in 32 countries, assessed both individual and group effects. There was a positive cross-population association between average rise in blood pressure with age and average levels of salt intake (measured by urinary sodium excretion) across 52 population samples in 32 countries at the group level, reflecting broad-scale population differences, and a positive relationship between urinary sodium excretion and blood pressure at the individual level (Elliott et al. 1996b). In a mortality study of cohorts of individuals from six U.S. cities, a positive association of mortality with measures of particulate matter pollution was found across those cities, adjusting for averaged site (city) effects derived from smoking, socioeconomic factors, and other potential confounding data measured at individual level (Dockery et al. 1993).

In the future, developments in exposure biomarkers (Hulka et al. 1990) and molecular epidemiology should lead to improved exposure assessment methods with increased specificity and accuracy. Although it will not be feasible to apply these methods to large numbers of people, collection of such data on small subsamples of the population will aid in validation of the exposure model and provide information on within-area variability in the exposure data and potentially on confounders. This may reduce bias and provide improved risk estimates, and hence strengthen any causal inferences (Guthrie and Sheppard 2001).

One of the opportunities presented by GIS technology is the adaptation of traditional study designs to the spatial context. For example, one of the most vexing problems for epidemiologists occurs when both the disease and environmental exposure under investigation are rare. Both the case–control and the cohort approach are likely to be costly and/or difficult because of issues of representativeness and sample size. As an alternative, hybrid designs have been used: the nested case–control (Paddle 1981) or the case–cohort study (Kupper et al. 1975), or more complex approaches such as two-stage sampling with oversampling of both exposed and diseased individuals (Rothman and Greenland 1998). This, too, can be cumbersome and costly.

GIS technology may offer a more efficient and cost-effective solution, at least for exposures that can be readily characterized geographically (Wartenberg 1994). With this approach, a nested case–control or case–cohort study can be conducted within a large-scale population-based cohort by specifying a geographic subset of the cohort with high relative exposure, on average, for direct study. For example, epidemiologic studies of the possible association between exposure to magnetic fields and the incidence of childhood leukemia have been limited by the low prevalence of high exposures because the higher exposures are relatively rare and widely dispersed: less than 10% of children with exposures above even twice the average background, less than 3% above three times, and less than 1% above four times the average background exposures (Ahlbom et al. 2000; Greenland et al. 2000; Zaffanella 1993). Case–control studies have consequently ended up with few children with high exposures and no obvious high-exposure cohort. The resulting small quantitative difference between exposed and unexposed individuals in these studies has limited their sensitivity and ability to yield a consistent and conclusive result (Wartenberg 2001).

In a demonstration project, a cohort of children with a far higher likelihood of being exposed to high levels of magnetic fields was identified using a geographically defined population living within 0.5 miles of a high-voltage electric power transmission line (Wartenberg et al. 1993, 1997). Because of the relatively low population density in the entire study region (New York State), results were of limited sensitivity, though modification and improvements to this design approach look promising.

## Conclusions

Advances in GIS and statistical methodology together with the availability of high-resolution, geographically referenced health databases present unprecedented new opportunities to investigate the environmental, social, and behavioral factors underlying geographic variations in disease rates at small-area scale. Such studies must be guided by good questions, excellent statistical methodology, and sound epidemiologic principles, including taking proper account of problems of data quality and the potential for bias and confounding. Spatial epidemiologic studies will become increasingly common in the future, both because of the instant visual appeal and wide availability of the new geographic techniques, and the desire for cleaner and healthier environments. With ongoing improvements in the data and methodologies, these studies will play an increasingly important role in our understanding of the complex relationships between environment and health.
